# An evaluation of transfer learning models in EEG-based authentication

**DOI:** 10.1186/s40708-023-00198-4

**Published:** 2023-08-03

**Authors:** Hui Yen Yap, Yun-Huoy Choo, Zeratul Izzah Mohd Yusoh, Wee How Khoh

**Affiliations:** 1 Faculty of Information Science and Technology, Multimedia University (MMU), Melaka, Malaysia; 2 Faculty of Information and Communication Technology, Universiti Teknikal Malaysia Melaka (UTeM), Melaka, Malaysia

**Keywords:** Authentication, Brainwaves, Transfer learning, Deep learning, Electroencephalography, EEG

## Abstract

Electroencephalogram(EEG)-based authentication has received increasing attention from researchers as they believe it could serve as an alternative to more conventional personal authentication methods. Unfortunately, EEG signals are non-stationary and could be easily contaminated by noise and artifacts. Therefore, further processing of data analysis is needed to retrieve useful information. Various machine learning approaches have been proposed and implemented in the EEG-based domain, with deep learning being the most current trend. However, retaining the performance of a deep learning model requires substantial computational effort and a vast amount of data, especially when the models go deeper to generate consistent results. Deep learning models trained with small data sets from scratch may experience an overfitting issue. Transfer learning becomes an alternative solution. It is a technique to recognize and apply the knowledge and skills learned from the previous tasks to a new domain with limited training data. This study attempts to explore the applicability of transferring various pre-trained models’ knowledge to the EEG-based authentication domain. A self-collected database that consists of 30 subjects was utilized in the analysis. The database enrolment is divided into two sessions, with each session producing two sets of EEG recording data. The frequency spectrums of the preprocessed EEG signals are extracted and fed into the pre-trained models as the input data. Three experimental tests are carried out and the best performance is reported with accuracy in the range of 99.1–99.9%. The acquired results demonstrate the efficiency of transfer learning in authenticating an individual in this domain.

## Introduction

The research’s interest in cognitive authentication has shown an increasing trend in recent years where it utilizes the cognitive state of individuals as the primary basis for authentication. The researcher communities believe that it could serve as an alternative for more conventional personal authentication methods as brain signals have specific characteristics that absent in most commonly utilized authentication methods. These characteristics are more privacy-compliant, unique and harder for an imposter to capture from a distance, hence increasing their resilience against spoofing attacks.

The brain signals of an individual can be acquired through a variety of techniques, including electroencephalography (EEG), electrocardiography (ECG), and electrodermal response (EDR). EEG is one of the methods which able to record the electrical activity that originates from the brain. It is a direct and the simplest noninvasive method to record brain electrical activity as it only places electrodes on the scalp’s surface [1]. The recorded EEG signals have low spatial resolution and poor signal-to-noise ratio that could be easily contaminated by noise and artifacts [2]. Moreover, the recorded signals are non-stationary, which indicates the signal’s characteristics change with time. Hence, sophisticated data analysis is necessary to retrieve useful information related to specific tasks from the EEG raw signals. With the ongoing exploration of EEG-based authentication, various machine learning approaches have been proposed and implemented, with deep learning being the most current trend [3, 4]. This approach has received widespread attention, specifically, the increasing adoption of convolutional neural networks (CNN) in the image classification domain due to its promising performance [5]. Deep learning is a kind of machine learning inspired by the structure of the human brain. The deep model is designed to extract significant and discriminative features from the input data by iteratively altering the data through different layers and predicting them accordingly.

In the EEG context, deep learning simplifies the data processing procedures by enabling an automatic learning style from preprocessing, and feature extraction to the classification phase [6]. Theoretically, deep learning is capable of achieving better feature extraction and more accurate pattern classification. However, training a deep learning model with millions of parameters requires vast input data and extensive computational resources [7]. The training and testing data in the field of signal processing cannot be expected to have the same probability distribution as different subjects on the same task may generate different features throughout different sessions. Moreover, the amount of EEG training data available for the various Brain Computer Interface (BCI) tasks is limited. Deep learning models that are trained with a small dataset may experience an overfitting problem [8]. Overfitting happens when a model fits perfectly against its training data and lacks of generalization ability to make accurate predictions on new data. Some of the well-known CNN architectures, e.g. Alexnet, includes dropout layers to minimize the effect of overfitting. Alternately, researchers have increasingly employed transfer learning to address the aforementioned issue in recent years. It is a technique in which a previously trained model, coined as pre-trained model, is used as the basis for a model on a new targeted problem, in our case, an EEG domain [7, 9]. It is practical and positively impacts various domains where it is difficult to increase performance due to the lack of training data [10]. When applied to a new task, transfer learning provides much better performance than training the model from scratch with little data.

Based on the existing EEG authentication studies, the exploration of different transfer learning models is limited. Although transfer learning research has been greatly conducted in different EEG domains, most reported results have focused on solving binary problems. It is necessary to perform a multi-class classification when it comes to user authentication problems. The effectiveness of using transfer learning in EEG-based authentication remains an open question. It indicates a critical research gap that has to be filled by more experimental investigations. This study describes a state-of-art EEG authentication system based on deep learning for recognizing an individual. The features extracted from EEG data are learned through multiple transfer learning models. The contributions of this work are two-fold. First, the feasibility of transferring pre-trained models’ knowledge to a new EEG domain is explored to address the limited sample size. Within the investigation, the knowledge of six pre-trained CNN models is transferred to adapt the EEG domain respectively. Second, beyond merely applying transfer learning to the EEG domain, comprehensive experimental analyses and in-depth performance evaluations are provided.

The rest of this paper is organized as follows: Sect. 2 begins with an overview of related work, followed by Sect. 3, which presents the methodology and the details of CNN models. Section 4 contains the results of conducted experiments. Section 5 discusses the major findings of conducted experiments. Finally, the conclusion is presented in Sect. 6.

## Related work

Authentication is a crucial component of any security system because it ensures that only authorized users have access to sensitive data. Several authentication methods have been developed over time in response to evolving security threats. These range from conventional password-based approaches to more sophisticated biometric. Password-based authentication is one of the conventional-based approaches which allow users provide a username and password to gain access to a system or application. It is simple to use as users just need to remember a combination of characters and enter their username and password on a login page. If the credentials match, access is granted. However, many users choose weak or easily guessable passwords making them susceptible to brute-force attacks or password guessing [11]. Moreover, some of the users reuse passwords across multiple accounts, creating a security risk. If one account is compromised, all accounts using the same password become vulnerable. Therefore, authentication systems based on biometric recognition technologies have received increasing attention from researchers in recent years. Biometrics refers to the recognition of individuals based on their physiological and behavioral characteristics [12]. Those characteristics are unique and measurable which can be used to label or describe individuals. In biometric modalities, physiological characteristics are related to the shape of the body such as fingerprint, palm veins, face, DNA, palm print, hand geometry, iris, and retina. Behavioral characteristics are related to a person’s behavior pattern, which is generally hard to copy and imitate. Some examples of behavioral biometrics are the way an individual signs their signature, voice printing, keystroke, and gesture. The process of biometric authentication involve capturing the physiological or behavioral characteristics, preprocessing the captured data, extracting relevant features, and comparing them with stored templates or reference data to authenticate or identify individuals. The specific algorithms and techniques used may vary depending on the biometric modality and the level of security required. For example, fingerprint authentication is a physiological method that involves capturing a user's fingerprints using a fingerprint sensor. The unique ridge patterns and minutiae points present in the fingerprints are detected and used for further processing. Feature extraction algorithms are then applied to extract relevant information from the captured fingerprint, and matching algorithms are used for fingerprint recognition. On the other hand, behavioral authentication encompasses various methods, and one common example is signature recognition. In this process, the user's signature is captured using a digital tablet and stylus pen. The obtained signature is then subjected to feature extraction techniques to identify and extract the distinctive characteristics of the signature. Pattern matching algorithms are employed to analyze and compare these unique signature features for authentication purposes. Although physiological and behavioral biometric modalities offer enhanced security compared to traditional authentication methods, they still face a prevalent issue of spoofing, in which an attacker creates a fake sample to circumvent the authentication system [13, 14]. For example, an attacker can construct a fake fingerprint or use a sample of recorded speech to impersonate another person. This emphasizes the need for more secure and trustworthy biometric authentication methods. Cognitive authentication offers a potential solution to this problem. Brain signals are unique to each individual and are difficult to replicate, making it difficult for attackers to spoof the system. Furthermore, brain signals are more private and difficult to be captured by an imposter from a distance, thus increasing their resistance against spoofing attacks.

With the maturity of computer technology and brain science, BCI has emerged as an essential research topic for exploring the communication between the human brain and computer or digital devices [15]. Applications BCI can observe users’ states or let them convey their intentions. In the meantime, the users’ brain signals are recorded and transmitted to a computer system for further data analysis. Afterward, the result is turned into a command, and the system is given instructions to carry out the desired activity. In the past decade, BCI has focused mainly on the medical field, helping patients with extensive paralysis, severe neuromuscular disorders, or loss of limbs regain some motor or communicative control [16]. EEG offers a noninvasive solution for BCI systems among different ways of acquiring brain signals. Researchers can record the electrical activities of the human brain by applying electrode sensors to the human scalp. This approach is widely studied because it is relatively convenient and reduces the user risk involved in brain signal acquisition [17, 18]. The combination of biometric authentication and brain science has the potential to improve security systems by leveraging physiological and cognitive characteristics that are unique to each individual. This interdisciplinary approach creates new opportunities for trustworthy and secure authentication methods. In general, an EEG-based authentication system consists of 5 major processing steps. First, the experiment needs to record users’ brain activity, which can be collected through EEG recordings using designed protocols such as stimulation, resting state, motor imaginary, and non-motor imaginary [19]. Second, the recorded data should be preprocessed to remove undesired artifacts as it could deteriorate the designed method’s performance. Third, feature extraction techniques are applied to preprocessed data to retrieve meaningful information. The next step is classifying the unlabeled data into one of the identified classes. Lastly, the final result is given to the user for decision-making.

Several methods have been employed to classify users based on their brain signals [20–22]. Traditional approaches often involve the use of shallow classifiers such as Linear Discriminant Analysis(LDA), Support Vector Machine (SVM) and K-nearest neighbor (k-NN). LDA is a popular linear classification technique that seeks to identify a linear combination of features that maximally separates classes [23]. SVM, on the other hand, is a potent binary classifier that uses a kernel function to handle non-linear separable data and finds an optimal hyperplane to separate data points into distinct classes. k-NN is a non-parametric algorithm that uses a distance metric to assign a new data point to the majority class among its k nearest neighbors. LDA tends to be effective when the classes are clearly differentiated and the data follow to the assumptions of linearity and equal class covariances. As for SVM, due to its ability to utilize kernel functions, it is especially effective when dealing with complex and non-linearly separable data [24]. k-NN, on the other hand, could be more adaptable and robust to complex and non-linear data, but it may be sensitive to the selection of value k and computationally costly, particularly with large datasets. They can typically accomplish good classification when the classification border is clear; nevertheless, it may produce unsatisfactory results if EEG signals are highly time-varying and contain a great deal of hidden and inconspicuous information [4]. Moreover, they are some challenges raised by the nature of EEG signals. First, EEG signals do not provide good spatial resolution on the human scalp due to the physical dimension of the surface electrodes and the dispersion of the signals generated by the sources on the cortex [25]. Besides, the signal-to-noise ratio of EEG is poor and might include high noises during EEG acquisition. In addition, EEG is a non-stationary signal whose statistical characteristics change with time [26]. Variations of the signal properties could happen if the EEG data of the same user is acquired at different sessions. Therefore, the development of robust algorithms capable of recognizing individual differences in EEG signals is required [27].

In recent years, deep learning has demonstrated its capability to assist in processing EEG signals. Deep learning enables computational models which consist of multiple layers to learn data representations with levels of abstraction. Previously, this technique has been implemented in processing complex data such as text, images, and audio signals [28], where the reported results are promising. In contrast to traditional linear classifiers such as SVM and LDA, which presume linear separability, deep learning models can effectively manage complex, non-linear relationships in EEG signals. This allows them to capture more complex patterns and enhances their classification performance. Moroever models based on deep learning have demonstrated robustness to variability in EEG signals. They can learn from diverse data and generalize well to unknown instances, making them more adaptable and able to handle different sessions and users. In the EEG context, it is able to derive discriminative features from the raw data and simplify the data processing steps, enabling an automatic learning style from preprocessing and feature extraction to classification while still preserving its competitive performance on specific tasks [6].

In [29], the authors initiated the use of deep learning and proposed 7 classifiers based on the CNN for the P300 speller application. Four single classifiers with diverse feature sets and three multi-classifiers made up the different models. These models were examined and compared on data set 2 from the third BCI competition. Prior to classification, the EEG raw data of 2 subjects were downsampled and bandpass filtered between 0.1 and 20 Hz. The findings demonstrated the potential of deep learning in recognizing EEG signals, and the best result was achieved using the proposed multi-classifier model, with a recognition rate of 95.5%. CNNs also have been employed in classifying motor imagery [9], where the authors combined extracted frequency, time, and location information from EEG recording signals. The results showed that the proposed deep learning methods could provide better classification compared to other approaches. In [30], the study proposed an epilepsy seizure prediction system on 24 subjects. The authors employed CNN for feature extraction and utilized the Support Vector Machine (SVM) for classification. The presented results were promising, with an average specificity of 90.8% and a sensitivity of 92.7%, respectively.

Other than the aforementioned domains, a study also explored the feasibility of using CNN to analyze EEG signals for user authentication [31]. Low frequency Steady-state visual evoked potentials (SSVEP) signals from 8 subjects with 2 sessions are extracted for further analysis. The CNN design of this study was based on the Shallow ConvNet structure proposed by [32]. Some adjustments on kernel size and the number of filters were applied to fit with the collected data. The results suggested the potential of EEG-based authentication based on SSVEP data and the CNN model. Besides using visual stimulation protocol, a personal identification system was proposed in [33] to classify resting state EEG signals using CNN. Data augmentation was performed on a public database called Physionet EEG Motor Movement. Only two resting states’ sessions from the public database were selected that are 1-min with eye-open and 1-min with eye-closed. The findings showed that the proposed method has offered some advantages in acquisition time and computational complexity. In [34], a Long Short Term Memory (LSTM) was applied. LSTM is a modified version of recurrent neural networks (RNN), making it easier to remember past data in memory. The authors combined event-related potential (ERP) features and SSVEP in authenticating an individual. The EEG data was collected from 20 subjects with 2 sessions separately. The results reported that the proposed method achieved a high verification accuracy rate of 91.44%.

The reported studies have demonstrated its capability in feature extraction and classification of CNN, in which the obtained results could be better than shallow architecture. However, their application to EEG-based authentication presents unique challenges that have to be addressed. To attain optimal performance, an adequate amount of labeled training data is required, posing a significant obstacle. Deep learning models are highly capable of capturing complex patterns and representations, but they require a large labeled dataset. Undoubtedly, more convoluted architecture can extract more discriminative features, but when there is inadequate training data, it frequently results in an overfitting issue that can even degrade its classification performance [7]. Overfitting happens when a model fits perfectly against its training data and lack of the generalization ability to make accurate predictions on new data. Due to the difficulty in collecting EEG data under different acquisition protocols, the sample size used to train deep learning models varied significantly across studies.

In order to cope with this issue, transferring learning (knowledge) from a pre-trained model can be an alternative in EEG-based authentication. It is a powerful technique as it has the ability to recognize and apply the knowledge and skills learned in previous tasks to a new domain with limited training data [35]. Transfer learning has been implemented in numerous domains, including image recognition [36, 37], language translation [38], biometrics [7, 13], and medical system [39]. Despite its advantages, the use of transfer learning in EEG signal processing is still limited, and the reported results of studies in the EEG domain were mostly achieved for binary classification [40–42]. When it comes to user authentication problem, multi-class classification are usually considered to classify the data to the respective user identity. Additionally, some well-known CNN models in other areas that transfer to the EEG domain are yet to be explored intensively. Therefore, this study attempts to fill in the gap by looking deeper into the implementation of CNN pre-trained models for EEG authentication.

## Methods

**Fig. 1:**
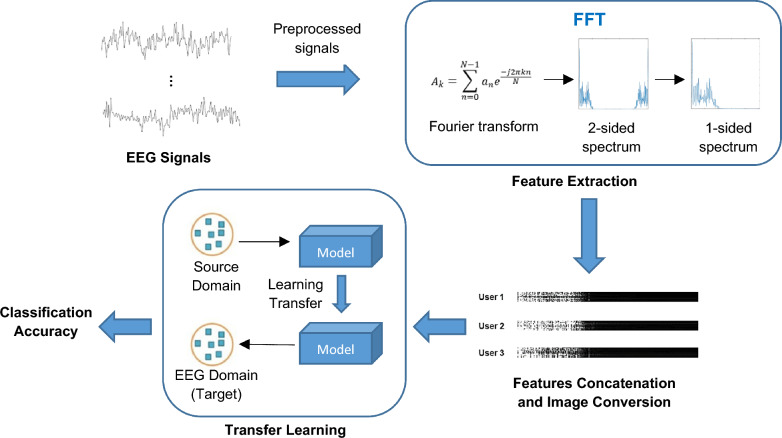
Overview architecture of proposed scheme

The overview architecture of the proposed system is illustrated in Fig. 1. First, the data acquisition protocol to capture an individual’s EEG signals is outlined in this section. The raw signal is preprocessed and segmented into a similar length trial. Following that, the feature extraction that employs Fast Fourier Transform (FFT) to extract the feature on the segmented EEG trials is described in Sect. 3.3. Section 3.4 describes the pre-trained CNN models that were adopted in this work. Experimental results of six different pre-trained CNN models are presented and discussed in Sect. 4 to determine how the transfer learning reacts to the EEG features. Discussion of the experimental results is presented in Sect. 5. Lastly, Sect. 6 is devoted to the conclusions of this work.

### Data acquisition protocol

EEG signals can be acquired using different designed protocols such as resting state, motor imaginary, non-motor imaginary, and stimulation protocol. As a way that enables participants to be more focused and their cognitive state to be more controllable by the experimenter throughout the data acquisition process [2, 19], the stimulation protocol is adopted in this study. A wireless consumer-grade device is employed rather than the cumbersome wired research-grade EEG devices to acquire EEG data. It is believed that this method would be more applicable in daily life, and user experiences during the EEG recording process would also be enhanced [43]. Thirty healthy volunteers, ranging in age from 18 to 39 years old have been recruited for this experiment. The EEG data were collected at a sampling rate of 256 Hz using an Emotiv EPOC + wireless headset. The device has 14 integrated electrodes and 2 reference sensors where each sensor is placed in the standard positions of the International 10–20 systems.

**Fig. 2:**
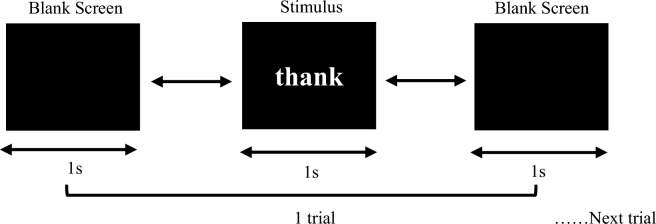
Visual stimulation design

Before the acquisition process, a brief introduction was given to the subjects. The study’s goal was briefly explained and a formal consent was obtained from each subject before the data recording process. They were instructed to avoid making large movements because it could affect their EEG readings. The entire process took place in a standard enclosed room. The stimulation design was based on research conducted by [19] in which the authors were motivated from a previous work [44] and generated strings and divided them into four categories: acronyms, low-frequency words, high-frequency words, and pseudowords. In order to evaluate the consistency of EEG data throughout multiple sessions, the acquisition process was separated into morning and afternoon sessions. In each session, the subject was presented with two sets of 120 single-word stimuli; the first set was presented in sequential order according to the wording category, while the second set was presented in a randomized mode. The individual was instructed to focus on and interpret each word in complete silence, with no large body movements permitted. As the subject’s semantic memory may have distinctive biometric characteristics, the emphasis is all on word presentation. The sample of Inter-Stimulus Interval (ISI) setting was shown in Fig. 2.

The 2 sessions yielded a total of four well-collected datasets, and the notations of each dataset are as follows:

Session 1:

- Sequential Dataset, *S1*_*S*_
- Randomized Dataset, *S1*_*R*_

Session 2:

- Sequential Dataset, *S2*_*S*_
- Randomized Dataset, *S2*_*R*_

### Preprocessing and segmentation

**Fig. 3:**
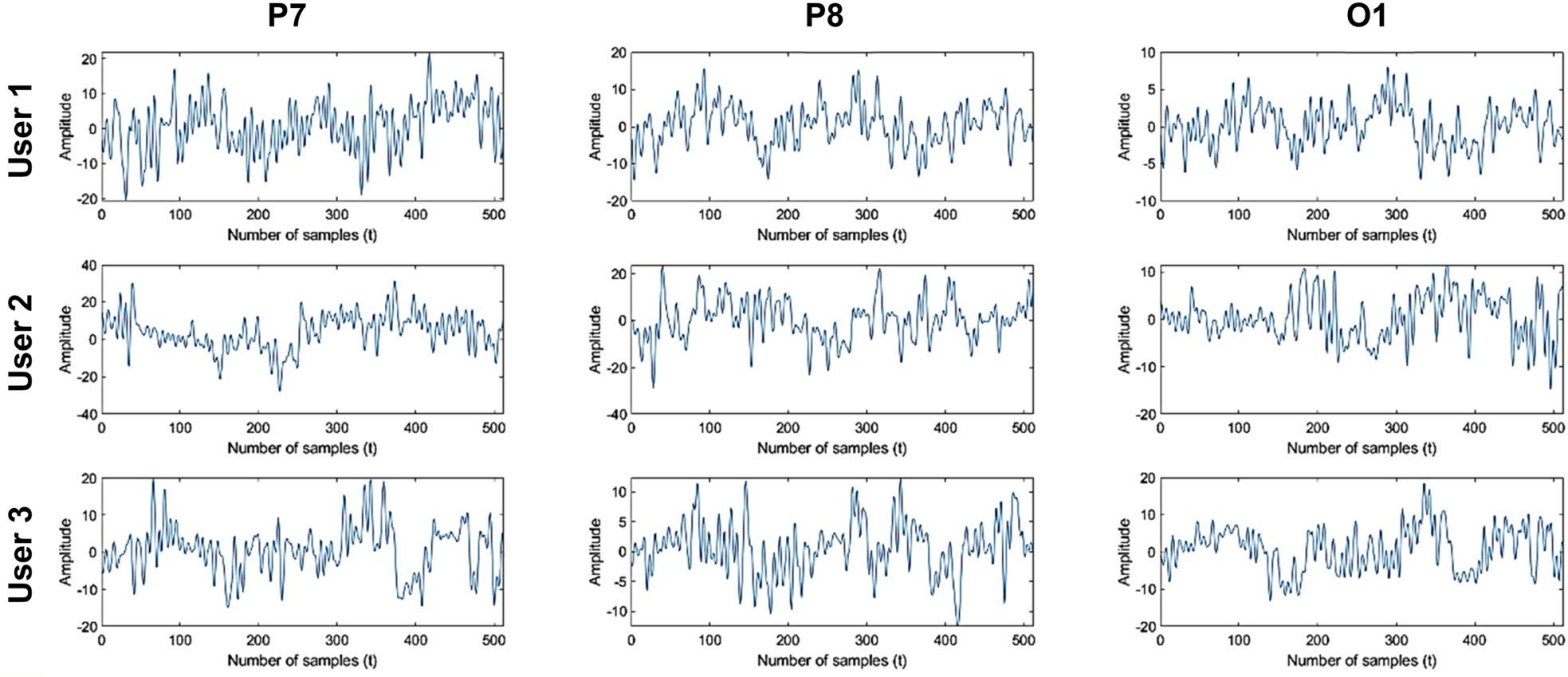
Sample of trials of different EEG channels of three different users

The collected datasets were preprocessed prior to the extraction of the appropriate features. First, a bandpass filter from 1 to 55 Hz was applied to filter continuous EEG data. The data was recorded from a total of 14 different EEG channels, capturing electrical activity from various regions of the brain. Next, the ocular artifacts that can interrupt the EEG data were removed with the help of Automatic Artifact Removal (AAR), one of the toolboxes available in the EEGLAB plugin. After preprocessing, the preprocessed data were epoched, and Event-Related Potentials (ERP) were created for each stimulus from -1000 ms prior to stimulus onset to 1000 ms following probe onset. Epoch rejection was further performed on the epoched data to eliminate unwanted artifacts. As a result, 100–120 trials were collected from each channel of the recorded data with a fixed duration of 2000 ms or 512 sample points (sampling rate = 256 Hz). Figure 3 illustrates the sample of segmented trials obtained from three different EEG channels (column) of three different users (row) from the database.

### Feature extraction

A time-dependent raw EEG signal fluctuates and contains noises unintentionally captured during the recording process. Instead of directly feeding the raw EEG signal to the next stage for classification, extracting more stable, informative and discriminative statistical features from the signal of interest is crucial. In this work, a fast Fourier Transform (FFT) as one of the established and proven techniques for extracting features from EEG signals [45] is adopted. This technique converts the EEG signal from the time domain into the frequency domain where the hidden features can become visible. In fact, the FFT algorithm was introduced based on the Discrete Fourier Transform (DFT). However, its signal transformation capability is faster than DFT due to the reduction in the looping process [46].

Given that a time domain EEG signal of length $N$ is denoted as ${a}_{n}$ where $n=0, 1, \dots , N-1$, the steps to convert the signal into a frequency representation are as below:

Step 1: Compute the Fourier transform of the EEG signal, ${a}_{n}$. The DFT can be formulated as:

1 $${A}_{k}=\sum_{n=0}^{N-1}{a}_{n}{e}^{\frac{-j2\pi kn}{N}}$$

where the ${e}^{\frac{-j2\pi kn}{N}}$ in the equation called the primitive *N*th root of unity and the *k* is the frequency of particular harmonic. The obtained ${A}_{k}$ is the Fourier transform coefficients. It generates a complex number of two-sided frequency spectrum of the signal, in the form of $a+bi$, where $a$ and $b$ are the real number and imaginary number, respectively.

Step 2: Compute the absolute and even the value of the two-sided spectrum by its signal length, $N$ to obtain its real magnitude as follows:

2 $${A}_{k}=\left|\frac{{A}_{k}}{N}\right|$$

Step 3: Since the two-sided spectrum obtained from Step 2 is symmetrical in which the spectrum is constructed by positive half and negative half; thus, the information on the negative frequency is redundant. Hence, the two-sided spectrum is converted to single-sided spectrum by discarding the second half of the spectrum and multiplying all points by 2 as follows:

3 $${A}_{l}=2\times {A}_{k}\left(i\right)$$

where $$i=1,\dots ,\frac{N}{2}+1$$.

**Fig. 4:**
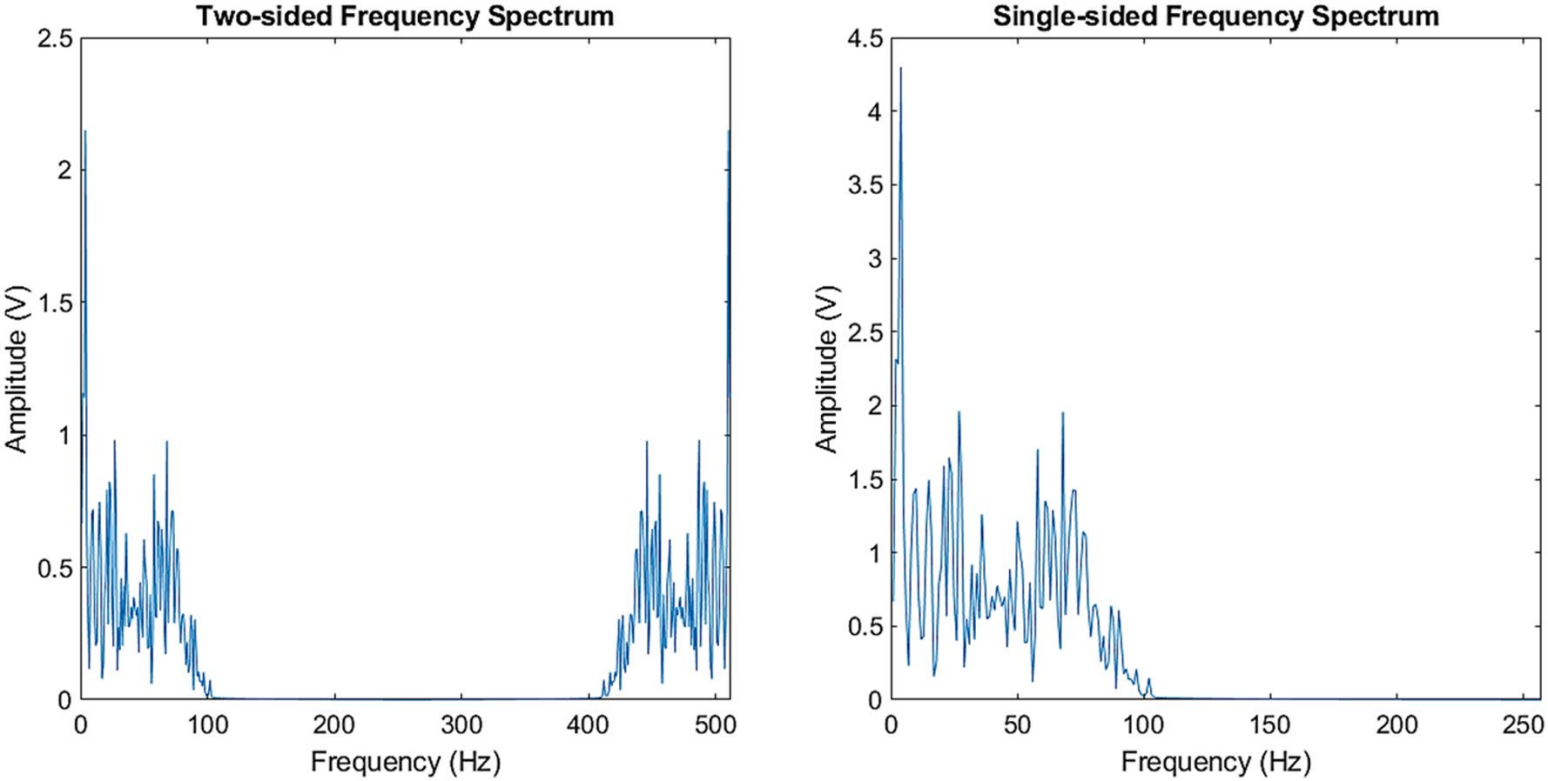
FFT features of EEG of two-sided frequency spectrum (left) and single-sided frequency spectrum (right)

As mentioned earlier, a trial contains 512 sampling points. The frequency spectrum after the FFT feature extraction generates a length of 257 sampling points. Figure 4 shows the examples of two-sided frequency spectrum (left) and single-sided frequency spectrum (right) of EEG data after the FFT extraction. It can be seen that the two-sided frequency spectrum is symmetrical to the single-sided spectrum with both halves are identical.

To be compatible with most of the pre-trained CNN models, all EEG channels are combined by vertically concatenating their FFT representations into a single matrix. This combination forms a 2-dimensional image as a feeding input to the models. This approach aims to maximize the accuracy of the results by combining the collective information from multiple channels. By merging the channels, we aim to capture global characteristics of brain dynamics and potentially enhance reliability and the discriminative power of the extracted features. This consolidation of channels into a unified FFT representation is motivated by the findings that combining channels from multiple scalp regions can provide a more informative input and lead to higher accuracy for the subsequent classification models [47]. The details are presented as follows:

The FFT features from all 14 channels are vertically concatenated to generate a matrix $X$, which is expressed as

4 $$X=\left[\begin{array}{c}{A}_{{l}_{1}}\\ {A}_{{l}_{2}}\\ \begin{array}{c}\vdots \\ {A}_{{l}_{m}}\end{array}\end{array}\right]$$

where ${A}_{l}$ is the obtained single-sided FFT features of an EEG signal, and the $m$ is the total number of EEG channels. The generated matrix has a size of $m\times n$,where the $n$ denotes length of FFT features. Lastly, to be compatible with most of the pre-trained CNN models, the matrix is converted into the image files which serve as the input images for the pre-trained models. This step enhances the compatibility and utilization of the pre-trained models, empowering them to leverage the merged FFT features for accurate classification.

**Fig. 5:**
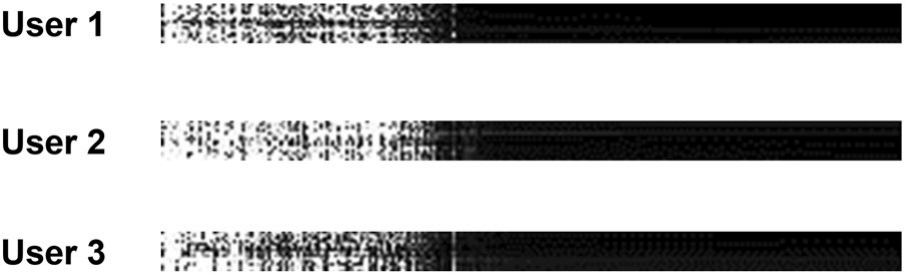
FFT matrix image for three different users

Figure 5 illustrates the sample images of extracted FFT features for three different users in concentenation process.

### Classification

#### Convolutional neural network

Classification is a supervised learning concept in machine learning that categorizes a set of data into classes. A reliable classification method is required to allow or deny a claimed user in authenticating process based on a given input. Various deep learning architectures have been implemented in the EEG domain, with CNN being the most prominent. It comprises multiple convolutional layers, each consisting of a series of filters known as convolutional kernels. These kernels are used to extract high dimensional features in which they are matrices that implement the dot product with the sub-region of input data and build feature maps in order to preserve information that is unique to the data. The computed feature maps are later transferred to the successive layer and performed in another round of feature extraction [48, 49].

When dealing with high-dimensional data, an activation function plays a crucial role as it can add non-linearity into neural network and help the network learn complex patterns. Technically, the activation function applies non-linear transformation over the input signal and determines which characteristics are utilized or omitted. The transformed output is subsequently supplied as input to the next layer. Nowadays, neural networks use a variety of activation functions. However, the Rectified Linear Unit (ReLU) is the most common. The key benefit of ReLU is that it does not activate all the neurons at once; instead, it only deactivates them when the linear transformation’s output is less than 0. Since it only activates a subset of neurons, the ReLU function is considerably more computationally efficient than other activation functions [50].

As the dimension of the generated feature map from the convolutional layer could be huge, the pooling layer is commonly used to reduce the dimension of the feature map. It is used to summarise the features contained in a region of the feature map, therefore reducing the number of parameters to be learned and the network’s computational load. In addition, the robustness of feature extraction could also be enhanced [51]. There are two typical types of operations on the pooling layer: average pooling and maximum pooling. Maximum pooling selects the highest value from the feature map, while average pooling computes the average value from the feature map. Typically, a CNN process begins with a number of convolutional layers, activation, and pooling functions. The final generated features are then fed into a fully connected layer. In this final layer, a softmax activation function is employed to calculate the probability of an input belonging to a specific class and drives the final result of classification.

#### Transfer learning

Since the training dataset in the EEG domain is restricted, transferring a pre-trained model that priorly learned from a large dataset for a specific task is an efficient technique to obtain acceptable accuracy with less training time and training samples [39, 52]. Although the EEG-based domain is different from the pre-trained models, which focus on object and image recognition, it is believed that the deep features learned by the best-performing pre-trained models will indeed perform well in EEG target domains. Thus, this work employs several pre-trained models that include GoogLeNet, InceptionV3, RestNet50, RestNet101, EfficientNet01, and DenseNet201with the purpose of improving the learning in the EEG target domain through the transfer of knowledge from the task that has learned by the models. In this section, the details of the architecture of each applied pre-trained model are described in depth.

#### GoogLeNet

GoogLeNet is an inception architecture that is also referred to as Inception-V1 that won in the ImageNet Large Scale Visual Recognition (ILSVRC) 2014. The GoogLeNet model has 22 layers when only layers with parameters are considered (or 27 layers if the pooling layer is considered). The primary goal of this architecture is to obtain high performance with low computational cost [53]. It introduced the novel inception block concept to CNN, which integrates multi-scale convolutional transformations: divide, transform, and merge. With this concept, the problem associated with learning different variations present in the same group of different images is resolved. Although the computational cost is optimized, its architecture could reduce the feature space in the next layer and may lead to the loss of relevant information [49].

Generally, these Inception models aim for parallel rather than deep layers, resulting in a wider rather than a deeper model. The basic module (Naïve) of the Inception V1 is composed of four parallel layers: 1 × 1, 3 × 3, 5 × 5 convolution, and 3 × 3 max pooling. The limitation of this model is that the 5 × 5 convolutional layer is computationally expensive. Therefore, a 1 × 1 convolutional layer is added before every convolutional layer. It results in faster computations and dimension reductions of the neural network [53].

#### Inception-V3

Inception-V3 is an improved version of Inception-V1 that aims to permit deeper networks while preventing the number of parameters from becoming excessively large. By achieving this, some modifications are done on the Inception-V3 model: the larger convolutions in the model are factorized into smaller convolutions, spatial factorization is applied to asymmetric convolutions, the Auxiliary classifier is used to enhance the convergence of very deep neural networks, and the grid size is reduced by expanding the activation dimension of the network filters. Despite having a deeper network (42 layers) than Inception-V1 and V2, the network’s speed has not been greatly impacted [54].

#### ResNet50 and ResNet100

Since a deep learning model contains a number of layers to solve complex problems, the accuracy levels may gradually degrade as the number of layers of the neural network increases. This deterioration in performance may result from the problem of vanishing or exploding gradients. ResNet was designed specifically to address this issue. It was introduced in 2015 under the name of Residual Network and won the ILSVRC2015 classification competition with a 3.57% error rate [55]. While Inception models concentrate on the width, ResNet focuses on the links between layers and adds a direct connection channel to the network. It is similar to Highway Network in that it allows original input information to be sent directly to the back of the layer. Hence, this neural network layer can learn without the whole output but instead learns the residual of the preceding network output [56]. ResNet introduced shortcut connections within layers to enable cross-layer connectivity. This method could speed up the convergence of the deep model and avoid gradient vanishing problem [49]. Although other studies have attempted to address the gradient vanishing problem, the ResNet algorithm stands out as a noteworthy solution. There are several ResNet versions with different convolutional layers while ResNet-50 and ResNet-101 are adopted in this study due to their promising results as reported in [55]. ResNet-50 is the version consisting of 50 layers, whereas ResNet-101 contains 101 layers. The architecture of both of these models can be found in [55].

#### EfficientNet-B0

EfficientNet, introduced by Tan and Le in 2019 [57], is one of the efficient models that achieves state-of-art accuracy on image classification tasks. The authors present a new scaling method that uniformly scales all the depth, width and resolution dimensions using a simple but effective compound coefficient. Given the fact that the model scaling does not modify layer operators of the baseline network, a good baseline network is necessary. Therefore, the authors built Efficient-B0, a mobile-size baseline, by performing a neural architecture search using an architecture similar to MnasNet [58] that optimizes FLOPS and accuracy. There are various scaling ratios for the models, for instance, EfficientNet-B1, B2, B3, B4, B5, B6, and B7. In this study, the baseline Efficient-B0 is chosen due to its architecture is the least complex and working on smaller images. The structure of EfficientNet-B0 can refer to [57].

#### DenseNet201

DenseNet, was developed by Huang et al. [59] as a continuation of ResNet in order to tackle the vanishing gradient problem. Besides, it is also used to overcome the limitation of ResNet. Although ResNet enables better information and gradient flow by directly adding the input to the output, a direct path is established from the previous layer to the current layer. However, simply adding up all the features might result in losing important information. DenseNet provides a solution that is more appropriate for this condition in which the authors used the concatenation concept and connected each layer to every other layer in a feed-forward fashion, allowing the output (feature maps) of all previous layers to be used as inputs into all subsequent layers [49]. Since DenseNet concatenates the previous layers’ features rather than adding them, feature reuse could be achieved as all previous layers’ feature maps are accessible. This approach could increase computational efficiency while also improve the information flow throughout the network. DenseNet is available in several versions, including DenseNet-121, DenseNet-169, and DenseNet-201, where the numbers represent the number of neural network layers in that particular model. In this research work, DenseNet-201 is adopted as it performed well in classifying different domains, including medical image recognition [52] and image-based drowsiness detection [60]. DenseNet-201 comprises four dense blocks with 6, 12, 48, and 32 convolution blocks, respectively. Between the dense blocks are transition layers that contain the following operations: batch normalization, convolution, and pooling. The architecture of the DenseNet-201 can be found in [59].

#### Transfer learning configuration

**Fig. 6:**
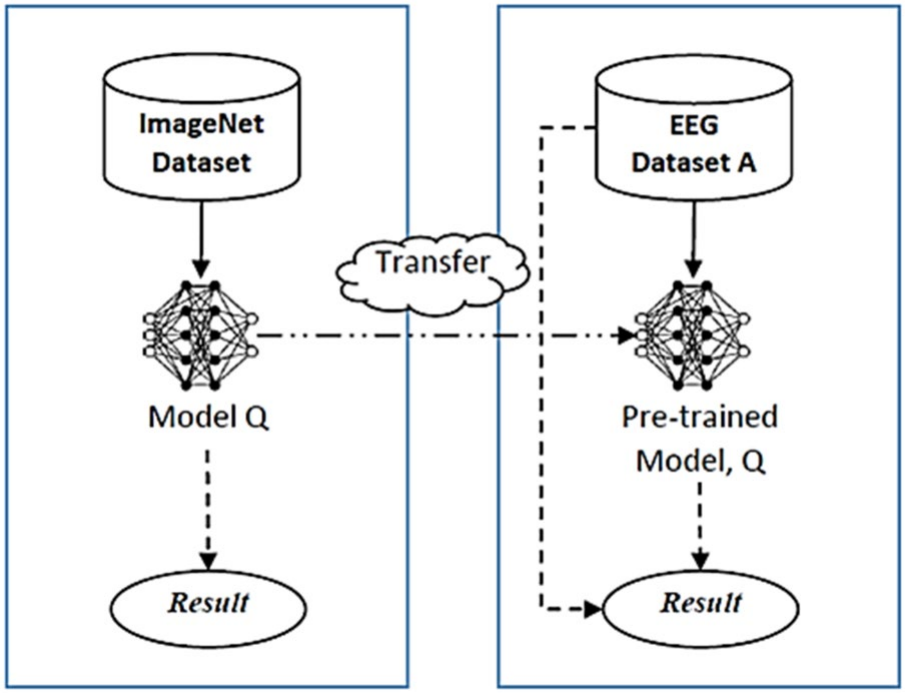
Transfer learning proposed model

Overall, the choice of GoogLeNet, InceptionV3, RestNet50, RestNet101, EfficientNet01, and DenseNet201 in this study are chosen based on their architectures in relation to spatial exploitation, depth, width, and multi-path [49]. The transfer learning model configuration is illustrated in Fig. 6. The selected CNN models, each was first trained on a large-scale dataset on a source domain, e.g. ImageNet dataset, and the knowledge (weights and biases) was transferred to EEG target domain. As illustrated in the figure, the pre-trained model denoted as Q was trained on the self-collected EEG dataset A. The dataset was split into training data and testing data with a ratio of M_A_:N_A_, where M_A_ is the ratio of training data and N_A_ is the ratio of testing data in dataset A, and M_A_ > N_A_. In this case, the ratio of 7:3 was used. The training set was used to train the model, whereas the test set was used to independently evaluate the model’s performance. Before incorporating these pre-trained models into the EEG domain, the final three layers were replaced with new settings consisting of a fully connected layer with 30 nodes (30 subjects leading to multiple classes), a softmax layer, and a classification output layer. For each pre-trained model, the architecture of the main model is preserved, as are the remaining parameters of the original model.

## Experimental analysis

### Settings

All the experiments were run using Matlab R2018a, and the computing systems were equipped with an i7-8700 core processor operating at 3.2 GHz, 64 GB of Random Access Memory (RAM), and the GPU specification as the NVIDIA GeForce GTX 1080Ti with 11 GB of video RAM. To match the data to the pre-trained models, the height and width of every image feature were rescaled to 227 by 227 pixels before sending into the input layer, with the exception of Inception-V3 where the images were resized to 299 by 299 pixels as the model’s requirement. The network’s initial learning rate was set to 0.01, and its minibatch size and maximum epochs were 32 and 30, respectively. It created up to 2910 maximum iterations at a rate of 50 each epoch.

In order to achieve consistent and fair performance results, each experiment was conducted five times for each dataset. Each time, a random selection of trials from the dataset was made for training and testing purposes. As noted in the preceding section, the data collection of this study consists of two sessions, with each session producing two sets of EEG recording data. In total, 4 sets of datasets were obtained: *S1*_*S*_, *S2*_*S*_, *S1*_*R,*_ and *S2*_*R*_. A series of experiments have been conducted to investigate the viability of employing pre-trained deep learning models in EEG-based authentication. The details are discussed in the subsequent section.

To comprehensively assess the performance of the classification on the imbalanced datasets, the performance metrics: accuracy, precision, specificity, sensitivity, and F1-score were calculated based on four parameters: true positive (TP), true negative (TN), false positive (FP), and false negative (FN), where they are generated as follows:

5 $$accuracy=\frac{TP+TN}{TP+TN+FP+FN}$$

6 $$precision=\frac{TP}{TP+FP}$$

7 $$sensitivity=\frac{TP}{TP+FN}$$

8 $$specificity=\frac{TN}{TN+FP}$$

9 $$F1 \,score=\frac{precision*sensitivity}{precision+sensitivity}$$

The outcomes that were found and reported in this paper include the averaged accuracy as well as the standard deviation (STD), which represents the degree of variation or dispersion around the average.

### Results

This section shows the experimental results and analyses of different pre-trained CNN models on the extracted EEG frequency signals. Using various combinations of EEG datasets, three experiments were conducted. Detailed descriptions of the experiments are provided in the following sections.

#### Experiment 1

**Fig. 7:**
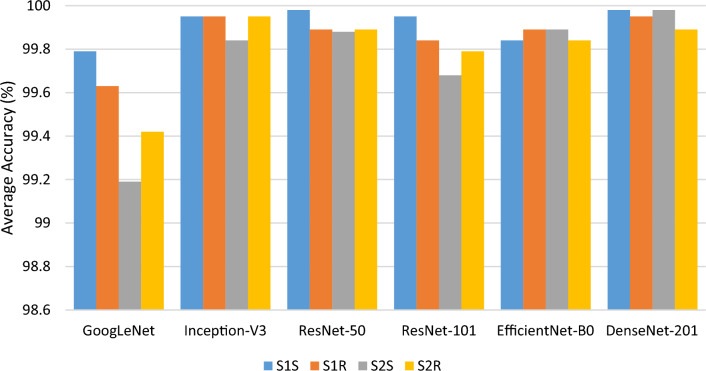
Summary of accuracy performances of Experiment 1 separated by different pre-trained models

The objective of the first experiment is to investigate the applicability of the individual EEG datasets derived using FFT transform to various pre-trained models, including GoogLeNet, Inception-V3, ResNet-50, ResNet-101, EfficientNet-B0, and DenseNet-201. Each dataset, including *S1*_*S*_*, S2*_*S*_*, S1*_*R*_*,* and *S2*_*R*_ was used to train and test six pre-trained models individually using a ratio of 7:3. Tables 1 and 2 show the classification performance of these six pre-trained models for both session 1 and session 2 datasets, respectively. As seen in the tables, both pre-trained models provided promising results, each achieving an accuracy above 99%. Based on the results of Session 1, ResNet-50 and DenseNet-201 had the best accuracy in the *S1*_*S*_ dataset at 99.98%, while Inception-V3 and DenseNet-201 attained 99.95% accuracy in the *S1*_*R*_ dataset. As for results in the Session 2, DenseNet-201 and Inception-V3 obtained the best accuracy performances with 99.98% and 99.95% among the pre-trained models in *S2*_*S*_ and *S2*_*R*_ datasets, respectively. Although these models achieve the best results, it is seen that the differences among other models are very trivial. Additionally, the small standard deviations among the reported performance measurements show the stability and consistency of the proposed method in user authentication. Figure 7 illustrates the summary of accuracy performances separated by different pre-trained models.

**Table 1:** Experimental results for *S1*_*S*_ and *S1*_*R*_ in the first session based on different pre-trained models

Model	Comparison of performance metrics (Averaged% ± Standard deviation)									
	Session 1: *S1*_*S*_					Session 1: *S1*_*R*_				
	Acc.	Pre.	Sens.	Spec.	F1	Acc.	Pre.	Sens.	Spec.	F1
GoogLeNet	99.79 ± 0.01	99.80 ± 0.002	99.79 ± 0.005	99.99 ± 0.001	99.79 ± 0.001	99.63 ± 0.08	99.63 ± 0.10	99.61 ± 0.07	99.97 ± 0.003	99.61 ± 0.08
Inception-V3	99.95 ± 0.08	99.95 ± 0.08	99.94 ± 0.08	99.99 ± 0.002	99.94 ± 0.08	**99.95 ± 0.08**	99.95 ± 0.07	99.94 ± 0.09	99.99 ± 0.002	99.94 ± 0.08
ResNet-50	**99.98 ± 0.01**	99.98 ± 0.01	99.98 ± 0.01	99.99 ± 0.002	99.98 ± 0.002	99.89 ± 0.002	99.90 ± 0.01	99.88 ± 0.002	99.98 ± 0.01	99.88 ± 0.003
ResNet-101	99.95 ± 0.08	99.95 ± 0.07	99.94 ± 0.09	99.99 ± 0.003	99.94 ± 0.08	99.84 ± 0.23	99.84 ± 0.22	99.82 ± 0.25	99.98 ± 0.002	99.83 ± 0.24
EfficientNet-B0	99.84 ± 0.08	99.84 ± 0.07	99.83 ± 0.09	99.99 ± 0.003	99.83 ± 0.08	99.89 ± 0.002	99.89 ± 0.01	99.89 ± 0.01	99.98 ± 0.002	99.89 ± 0.001
DenseNet-201	**99.98 ± 0.01**	99.98 ± 0.01	99.98 ± 0.01	99.99 ± 0.002	99.98 ± 0.01	**99.95 ± 0.08**	99.95 ± 0.07	99.94 ± 0.09	99.99 ± 0.003	99.94 ± 0.08

*ACC* Accuracy, *Pre* Precision, *Sens* Sensitivity, *Spec* Specificity, *F1* F1 score

The bold values indicate the highest accuracy

**Table 2:** Experimental results for *S2*_*S*_ and *S2*_*R*_ in the second session based on different pre-trained models

Model	Comparison of performance metrics (Averaged% ± Standard deviation)									
	Session 2: *S2*_*S*_					Session 2: *S2*_*R*_				
	Acc.	Pre.	Sens.	Spec.	F1	Acc.	Pre.	Sens.	Spec.	F1
GoogLeNet	99.19 ± 0.53	99.21 ± 0.53	99.17 ± 0.54	99.96 ± 0.02	99.18 ± 0.55	99.42 ± 0.23	99.42 ± 0.22	99.39 ± 0.24	99.96 ± 0.01	99.40 ± 0.24
Inception-V3	99.84 ± 0.23	99.85 ± 0.21	99.84 ± 0.23	99.98 ± 0.01	99.84 ± 0.23	**99.95 ± 0.08**	99.95 ± 0.28	99.94 ± 0.08	99.98 ± 0.003	99.94 ± 0.08
ResNet-50	99.88 ± 0.01	99.90 ± 0.002	99.90 ± 0.01	99.99 ± 0.02	99.89 ± 0.001	99.89 ± 0.002	99.90 ± 0.01	99.44 ± 0.63	99.99 ± 0.02	99.89 ± 0.002
ResNet-101	99.68 ± 0.15	99.69 ± 0.14	99.66 ± 0.14	99.97 ± 0.01	99.67 ± 0.14	99.79 ± 0.30	99.78 ± 0.31	99.78 ± 0.31	99.98 ± 0.01	99.78 ± 0.31
EfficientNet-B0	99.89 ± 0.16	99.87 ± 0.019	99.86 ± 0.20	99.98 ± 0.01	99.86 ± 0.19	99.84 ± 0.23	99.84 ± 0.23	99.83 ± 0.24	99.98 ± 0.01	99.83 ± 0.24
DenseNet-201	**99.98 ± 0.01**	99.98 ± 0.02	99.98 ± 0.02	99.99 ± 0.01	99.99 ± 0.01	99.89 ± 0.01	99.90 ± 0.01	99.88 ± 0.01	99.99 ± 0.02	99.89 ± 0.01

The bold values indicate the highest accuracy

#### Experiment 2

**Fig. 8:**
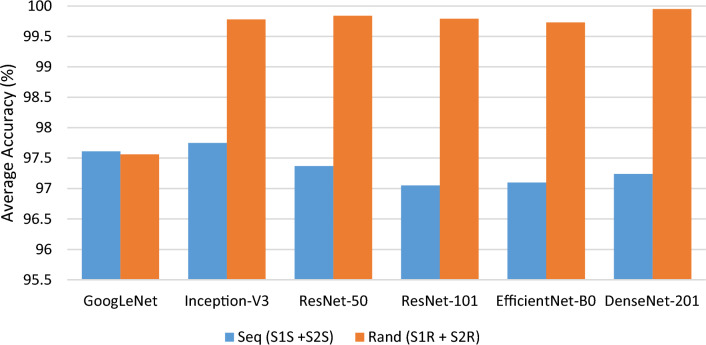
Summary of accuracy performances of experiment 2 (*Seq* and *Rand* datasets) separated by different pre-trained models

A second experiment was undertaken using a larger dataset to examine the classification performance and validate the previous experiment’s findings. The different session datasets were merged to create a new dataset known as *Seq* dataset (*S1*_*S*_ + *S2*_*S*_) and *Rand* dataset (*S1*_*R*_ + *S2*_*R*_). The training and testing procedure adhered to the 7:3 ratio and five runs were executed for a fair evaluation. Tables 3 and 4 summarise the performance of the pre-trained models in EEG classification with the merged datasets. Observed from the experimental results for *Seq* dataset, all pre-trained models obtained consistent results of around 97% accuracy, with Inception-V3 obtaining the best accuracy at 97.75%. On the other hand, the experimental results for *Rand* dataset gained close to perfect accuracy except for GoogleNet, with an accuracy of 97.56%. The DenseNet-201 is reported to have the highest accuracy of 99.95%. As compared with both *Seq* and *Rand* datasets, it is also found that the latter has slightly outperformed the former. It can be due to the randomize datasets (*S1*_*R*_ and *S2*_*R*_) are more consistent than the sequential datasets (*S1*_*S*_ and *S2*_*S*_). Again, the low discrepancies in the standard deviation of all the performance measurements demonstrate the consistency and stability of the method. Figure 8 shows the summary accuracy performances of both the *Seq* and *Rand* datasets separated by different pre-trained models.

**Table 3:** Experimental results for combined sessions- *Seq* (*S1*_*S*_ + *S2*_*S*_) based on different pre-trained models

Model	Comparison of classification accuracy (Averaged% ± Standard deviation)				
	Acc.	Pre.	Sens.	Spec.	F1
GoogLeNet	97.61 ± 0.34	97.93 ± 0.85	97.54 ± 0.34	99.91 ± 0.001	97.45 ± 0.23
Inception-V3	**97.75 ± 0.46**	97.80 ± 0.54	97.69 ± 0.46	99.92 ± 0.02	97.64 ± 0.47
ResNet-50	97.37 ± 0.08	97.30 ± 0.08	97.30 ± 0.08	99.91 ± 0.003	97.30 ± 0.08
ResNet-101	97.05 ± 0.23	96.97 ± 0.23	96.96 ± 0.23	99.89 ± 0.01	96.96 ± 0.23
EfficientNet-B0	97.10 ± 0.15	97.04 ± 0.15	97.02 ± 0.14	99.90 ± 0.01	97.01 ± 0.13
DenseNet-201	97.24 ± 0.27	97.16 ± 0.27	97.16 ± 0.27	99.90 ± 0.01	97.16 ± 0.27

The bold values indicate the highest accuracy

**Table 4:** Experimental results for combined sessions- *Rand* (*S1*_*R*_ + *S2*_*R*_) based on different pre-trained models

Model	Comparison of classification accuracy (Averaged% ± Standard Deviation)				
	Acc.	Pre.	Sens.	Spec.	F1
GoogLeNet	97.56 ± 0.27	97.76 ± 0.64	97.50 ± 0.27	99.91 ± 0.01	97.44 ± 0.20
Inception-V3	99.78 ± 0.08	99.79 ± 0.07	99.78 ± 0.07	99.98 ± 0.003	99.79 ± 0.07
ResNet-50	99.84 ± 0.08	99.84 ± 0.08	99.84 ± 0.07	99.98 ± 0.003	99.84 ± 0.08
ResNet-101	99.79 ± 0.08	99.79 ± 0.08	99.78 ± 0.07	99.98 ± 0.002	99.78 ± 0.08
EfficientNet-B0	99.73 ± 0.02	99.73 ± 0.01	99.73 ± 0.01	99.98 ± 0.002	99.73 ± 0.005
DenseNet-201	**99.95 ± 0.02**	99.95 ± 0.03	99.94 ± 0.01	99.99 ± 0.002	99.94 ± 0.002

The bold values indicate the highest accuracy

#### Experiment 3

**Fig. 9:**
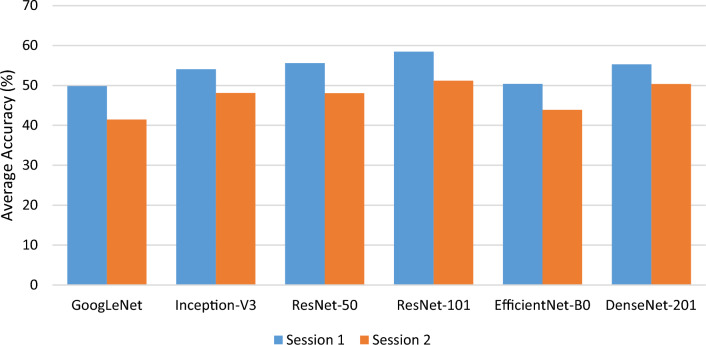
Summary of accuracy performances of experiment 3 (*Session 1* and *Session 2*) separated by different pre-trained models

This experiment aimed to assess the classification capability of EEG signals across different datasets within the same session in user authentication. There are two types of settings: the *S1*_*S*_ dataset was utilized to train the models, and the *S1*_*R*_ dataset was used for testing. Similarly, the second setting used *S2*_*S*_ for training and S2_R_ for testing. The classification performances of six pre-trained models are summarised in Table 5. The performances of both settings deteriorated dramatically compared to the previous two experiments. It was because the datasets used for training and testing were separated and thus, yielded low performances. It can be seen that the accuracy performance was degraded in the range of 41.45% to 58.48% for both settings. The ResNet-101 achieved the best accuracy performances in all sessions’ settings, with 58.48% and 51.19%, respectively. It is also observed that GoogLeNet yielded the worst performances. The accuracy performances of these experiments are also summarised and illustrated in Fig. 9.

**Table 5:** Experimental results for Session 1(*S1*_*S*_ + *S1*_*R*_) and Session 2(*S2*_*s*_ + *S2*_*R*_) based on different pre-trained models

Model	Comparison of performance metrics (Averaged% ± Standard deviation)									
	Session1: *S1*_*S*_*(training)* + *S1*_*R*_*(testing)*					Session 2: *S2*_*s*_*(training)* + *S2*_*R*_*(testing)*				
	Acc.	Pre.	Sens.	Spec.	F1	Acc.	Pre.	Sens.	Spec.	F1
GoogLeNet	49.84 ± 1.40	48.30 ± 3.39	49.76 ± 1.61	98.21 ± 0.05	49.67 ± 9.73	41.45 ± 0.52	39.49 ± 0.33	41.19 ± 0.50	97.91 ± 0.02	37.90 ± 0.03
Inception-V3	54.07 ± 0.20	50.87 ± 0.63	54.00 ± 0.35	98.36 ± 0.01	48.46 ± 0.76	48.11 ± 0.18	42.87 ± 0.83	48.08 ± 0.46	98.15 ± 0.01	42.80 ± 0.33
ResNet-50	55.59 ± 1.45	51.20 ± 2.67	55.72 ± 1.49	98.42 ± 0.05	49.90 ± 2.23	48.06 ± 0.02	42.98 ± 2.47	48.08 ± 0.09	98.15 ± 0.01	42.69 ± 0.41
ResNet-101	**58.48 ± 1.01**	51.95 ± 2.09	58.74 ± 1.11	98.52 ± 0.04	52.28 ± 0.85	**51.19 ± 0.67**	43.47 ± 0.35	51.23 ± 0.51	98.26 ± 0.02	45.43 ± 0.31
Efficient-B0	50.40 ± 0.43	47.37 ± 2.18	50.15 ± 0.41	98.23 ± 0.02	45.58 ± 0.73	43.91 ± 1.08	42.04 ± 0.58	44.14 ± 1.04	98.00 ± 0.04	40.39 ± 1.36
DenseNet-201	55.30 ± 1.59	53.21 ± 2.40	55.51 ± 1.50	98.37 ± 0.11	49.84 ± 1.08	50.38 ± 0.74	45.88 ± 4.55	50.40 ± 0.79	98.23 ± 0.03	45.11 ± 1.76

The bold values indicate the highest accuracy

## Discussion

Transfer learning has been exploited in this study. Several experimental tests were conducted to evaluate the performance of pre-trained models in authenticating an individual’s identity using EEG signals. Three experiments were conducted to assess the performance of the proposed method on different datasets. The overall results obtained in this study reveal that the multi-class classification problem can be well addressed by applying the transfer learning method. The self-collected database is relatively small. With the extracted FFT inputs, the proposed pre-trained models could learn effective features and achieve good classification results in experimental tests 1 and 2. Although GoogleNet produced lower results among the pre-trained models, the accuracies reported in experimental Test 1 and 2 were between 97.56% and 99.79%, which is within a promising range. It is speculated that the number of convolutional layers in GoogleNet is slightly insufficient for the task to identify the data to the correct classes as compared with other models. In the case of Inception-V3, the accuracy ranged from 97.75% to 99.95% in both experimental tests, respectively. In experimental Test 1, this pre-trained model performed well, achieving the greatest accuracy in the *S1*_*R*_ and *S2*_*R*_ individual datasets. Furthermore, it also exhibited the highest performance in the *Seq (S1*_***S***_ + *S2*_***S***_*)* dataset for experimental Test 2. Despite having a similar structure to GoogleNet, its improved concepts have proven its competitiveness against other advanced training models such as ResNets and DenseNet-201, which incorporate multiple paths and deeper layers. When comparing EfficientNet-B0 to other pre-trained models in terms of accuracy, it may exhibit slightly lower accuracy in specific sessions. It is relatively smaller and less complex which may limit its ability to detect and represent complex data patterns and characteristics. Nevertheless, despite these constraints, EfficientNet-B0 still demonstrates stable accuracy in the first experimental test where its accuracy ranged from 99.84% to 99.89% and in the second experimental test, from 97.10% to 99.73%. Overall, EfficientNet-B0 performs reliably in EEG-based authentication tasks. As can be observed from the results, the DenseNet-201 and ResNet-50 slightly outperformed the other pre-train models in terms of accuracy, precision, specificity, sensitivity, and F1-score. Consideration must be given to the architectures of these pre-trained models to comprehend why their outcomes are superior to those of other models. First, ResNet-50 consists of multiple residual blocks, each consisting of an identity mapping layer and shortcuts. This unique architecture enables an increase in precision with increasing layer depth. ResNet-101 is based on the same concept as ResNet-50 but with a larger number of layers. Although ResNet-101's performance is slightly lower than ResNet-50, it still demonstrates a good level of accuracy. On the other hand, DenseNet is an enhanced version of ResNet designed to overcome decreased performance caused by losing information due to longer paths between the input and output layers in the neural network. Using the concepts of concatenation and feature reuse, this architecture has complete access to the feature maps of all preceding levels, freeing the network of the need to relearn previously useful features. Therefore, it implies that the benefits of DenseNet are effective in recognizing EEG multi-class problems. Although it is reported that these models achieved the best results, the performance differences among other models are trivial. The contribution of other pre-trained models that achieve comparable outcomes cannot be neglected. Additionally, the minor differences in the standard deviations of all the reported performance measurements demonstrate the stability and consistency of the EEG features towards the pre-trained models that are capable of accurately classifying the identification of an individual.

In experimental test 3, the best performance in authenticating individuals was obtained by the ResNet-101 with an accurate rate of 58.48% and 51.19% in Session 1 and Session 2, respectively. The performance degradation and a significant drop in accuracy was observed from remaining models as well when the training and testing data were drawn from separate datasets. Consistent with the findings of experimental tests 1 and 2, GoogleNet, which has the fewest convolutional layers, obtained the lowest accuracy, followed by EfficientNet-B0. These results suggest that models with a lesser number of convolutional layers may have difficulty capturing complex patterns and features in the EEG signals, resulting in a decrease of accuracy. On the other hand, models with deeper architectures, such as ResNet-101, ResNet-50, and DenseNet-201, demonstrated greater accuracy, highlighting the benefits of employing deeper architectures for enhanced performance. Although Inception-V3 did not attain the highest level of accuracy, its performance was promising. In spite of its slightly lower accuracy than the best-performing models, Inception-V3 remains a viable option for EEG-based authentication tasks, especially when computational resources are restricted.

In addition to considering the model's architecture, it is essential to take into account the inherent challenges associated with EEG signals: poor signal-to-noise ratio and non-stationary nature. Therefore in order to develop a robust EEG-based authentication system, the classification model may require a method to train and adapt diverse input data from different datasets. By incorporating techniques for dealing with variations in signal quality, the model can enhance its ability to accurately classify and authenticate individuals based on EEG signals.

## Conclusion

This paper explored the effectiveness of transfer learning in EEG-based user authentication, and six pre-trained CNN models were adopted and compared. These pre-trained models consist of GoogLeNet, Inception-V4, ResNet-50, ResNet-101, EfficientNet-B0, and DenseNet-201which employed on the self-collected EEG database to classify the extracted FFT frequency features and perform multi-classes user recognition. Three experimental tests were conducted, and the results were analyzed and discussed. The highest accuracy of 99.98% was attained using the DenseNet-201 model to classify thirty subjects. Experiments demonstrate that without requiring CNN model training from scratch, the proposed pre-trained models are able to transfer relevant knowledge (weights and biases) to authenticate an individual. In addition, this study also assessed the test–retest repeatability of all the subjects. The results indicate a decline in performance when different datasets were utilized. In future work, the issue of repeatability over time can be further studied, and novel approaches that can train and adapt diverse input data from different datasets in authenticating individuals could potentially be explored.

## Data Availability

Not applicable.

## Abbreviations

AAR: Automatic Artifact Removal
BCI: Brain Computer Interface
CNN: Convolutional neural network
DFT: Discrete Fourier Transform
ECG: Electrocardiography
EDR: Electro-dermal response
EEG: Electroencephalogram
ERP: Event-related potential
FFT: Fast Fourier Transform
FN: False negative
FP: False positive
ILSVRC: ImageNet Large Scale Visual Recognition
ISI: Inter-Stimulus Interval
LDA: Linear Discriminant Analysis
LSTM: Long Short Term Memory
RAM: Random Access Memory
RNN: Recurrent neural networks
ReLU: Rectified Linear Unit
SSVEP: Steady-state visual evoked potentials
SVM: Support Vector Machine
TN: True negative
TP: True positive
